# Inflammation and psychopathology in children following PICU admission: an exploratory study

**DOI:** 10.1136/ebmental-2018-300027

**Published:** 2018-10-09

**Authors:** Giorgia Caspani, Georgina Corbet Burcher, M Elena Garralda, Mehrengise Cooper, Christine M Pierce, Lorraine C Als, Simon Nadel

**Affiliations:** 1 Department of Surgery and Cancer, Imperial College London, London, UK; 2 Centre for Psychiatry, Imperial College London, London, UK; 3 Department of Paediatric Intensive Care, St Mary’s Hospital, Imperial College Healthcare NHS Trust, London, UK; 4 Department of Paediatric Intensive Care, Great Ormond Street Hospital, London, UK

## Abstract

**Background:**

Survivors of critical illness in childhood commonly display subsequent psychiatric symptoms including emotional and behavioural difficulties, and manifestations of post-traumatic stress disorder (PTSD). Anomalies in inflammatory profiles are an established finding in these childhood psychiatric conditions.

**Objective:**

This exploratory study aimed to investigate whether abnormal peripheral blood inflammatory markers measured during paediatric intensive care unit (PICU) admission were associated with psychiatric symptoms after discharge.

**Methods:**

We performed a prospective observational cohort study on 71 children with septic illness, meningoencephalitis and other critical disorders admitted to two PICUs between 2007 and 2010. 3–6 months following discharge, subjects were assessed for global psychiatric risk (ie, presence of emotional and behavioural difficulties on the parental Strengths and Difficulties Questionnaire (SDQ)), and for PTSD risk using the child-rated Impact of Events Scale (IES-8). Inflammatory and related biological markers were transcribed from PICU admission notes (white cell count, lymphocytes, neutrophils, C reactive protein (CRP), platelets, fibrinogen and lactate).

**Findings:**

Global psychiatric risk at follow-up was associated with abnormal lymphocyte count during admission (χ^2^=6.757, p=0.014, n=48). In children with sepsis, partial correlation analyses controlling for age and gender highlighted associations between (i) SDQ scores and low lymphocyte count (r=−0.712; p=0.009, n=14), and (ii) IES-8 score and high CRP levels (r=0.823; p=0.006, n=11). These associations remained after correction for multiple comparisons.

**Conclusion:**

These results support the hypothesis that acute inflammation may play a role in determining the development of psychopathology following PICU admission.

**Clinical implications:**

If the findings are replicated, they may help to better highlight which children are at risk of post-PICU psychopathology and appropriately target follow-up.

## Background

The trauma of critical illness in children is a risk factor for the development of psychiatric sequelae, with 20%–30% of children presenting psychiatric symptoms after discharge from paediatric intensive care units (PICUs).^1^ Symptoms of post-traumatic stress disorder (PTSD) are particularly prevalent.^2^

Numerous inflammatory abnormalities have been widely established to have associations with a range of psychiatric and developmental disorders. Across the diagnostic categories, studies comparing patients with controls have shown that those suffering from psychiatric disorders have skewed inflammatory blood profiles favouring a pro-inflammatory milieu.^3–6^ Adolescent depression has been specifically linked to altered natural killer (NK) cell activity and increased levels of circulating pro-inflammatory cytokines (interleukin (IL)-1β and tumour necrosis factor-alpha (TNF-α).^7^ Abnormal lymphocyte counts have been proposed as a potential biomarker for a number of psychiatric illnesses^8^ and patients with PTSD have approximately twice the odds of C reactive protein (CRP) elevations than those without PTSD.^9^ Abnormalities in inflammatory and stress response profiles have also been observed in subjects with a history of childhood trauma.^10–14^ Therefore, it is possible that inflammation might be a mediator between childhood trauma and the development of psychopathology.

Further evidence supporting this finding is that children admitted to PICU with septic illness (which is associated with a state of overwhelming systemic inflammation^15^) have been found to present more severe PTSD symptoms compared with patients from other PICU illness subgroups who experienced lesser degrees of inflammatory response.^1^ In the assessment of the neuroendocrine stress response, altered cortisol profiles are associated with post-traumatic symptoms in children discharged from PICU compared with healthy controls.^16^ This finding supports a functional link between abnormal activity of the hypothalamic-pituitary-adrenal (HPA) axis (bidirectionally linked to the inflammatory immune response) and psychiatric symptoms at follow-up.

Inflammation and abnormal inflammatory biomarkers are central features of a number of critical diseases leading to PICU admission. In light of the findings from previous studies investigating the neuropsychiatric sequelae of critical illness and PICU admission, we hypothesised that degree of inflammation (measured by abnormal clinical biomarkers) would be associated with subsequent psychiatric symptom burden. The range of critical illnesses were classified into three different subtypes (septic illness, meningoencephalitis and other illnesses) to account for presumed differing degrees of inflammation, and to evaluate whether outcomes varied depending on systemic peripheral inflammation or localised cerebral infection. Inflammatory markers were expected to be most relevant for the psychiatric adjustment of children following systemic infections, to be of intermediary relevance for those with localised infection and least relevant for children with primarily non-infectious diseases.

This is, to the best of our knowledge, the first study addressing the association between inflammation and psychiatric sequelae in the context of critical illness in children and lays the foundation for further work in this field.

## Objective

To examine the association between inflammatory markers and psychiatric symptoms in subjects following PICU admission.

## Methods

### Participant characteristics and recruitment

This secondary analysis was performed on data collected for a previous study investigating the psychiatric symptoms of children 3–6 months following discharge from PICU.^1^ The adapted database contained clinical and demographic information on 71 children aged 4–16 years previously admitted to PICUs at St Mary’s and Great Ormond Street Hospitals in London, between 2007 and 2010. The cohort was a subsample from the full cohort (see ref.^1^) and was split by illness type into three groups; septic illness, meningoencephalitis and patients admitted with other critical illnesses.

During screening, information on the patients’ health history was collected via a questionnaire and those with medical histories indicating prior or concurrent psychiatric or neurological disorders or developmental delay were excluded from the study. Psychiatric difficulties prior to PICU admission were ascertained by parental report of emotional or behavioural difficulties (measured in comparison to children of the same age) and presence or absence of the requirement for related professional help. Clinical case notes were also checked for documentation of the presence of previous psychiatric diagnoses. Details on background information collected including demographics, past and acute clinical descriptors for the cohort are available in table 1. Details on illness characteristics were obtained from clinical cases notes. Illness severity was ascertained by reference to length of PICU admission, and paediatric index of mortality 2 (PIM2) score which incorporates values from a number of clinical observations (blood pressure, pupillary light reaction, PaO_2_, FiO_2_, capillary base excess, presence of mechanical ventilation and reason for admission) at point of admission to PICU and predicts outcome based on illness severity.^17^

**Table 1:** Patient demographic and general health status characteristics

	Full cohort % (n/N)	Meningoencephalitis % (n/N)	Septic illness % (n/N)	Other illnesses % (n/N)
Age on admission	9.54±3.65	10.52±3.73	9.50±3.50	9.00±3.67
Gender				
Male	63 (45/71)	84 (16/19)	39 (7/18)	65 (22/34)
Female	37 (26/71)	16 (3/19)	61 (11/18)	35 (12/34)
Socioeconomic status (NS-SEC level)*				
Level I	44 (27/61)	37 (6/16)	40 (6/15)	50 (15/30)
Level II	26 (16/61)	44 (7/16)	13 (2/15)	23 (7/30)
Level III	30 (18/61)	19 (3/16)	47 (7/15)	27 (8/30)
Ethnicity				
White	59 (42/71)	74 (14/19)	44 (8/18)	59 (20/34)
Other	41 (29/71)	26 (5/19)	56 (10/18)	41 (14/34)
Neonatal				
SCBU/NICU admission	16 (11/71)	26 (5/19)	17 (3/18)	9 (3/34)
General health*				
Chronic medical condition	30 (21/70)	26 (5/19)	17 (3/18)	39 (13/33)
Past health status (parent report)*				
Good	77 (54/70)	68 (13/19)	94 (17/18)	73 (24/33)
Intermediate/poor	23 (16/70)	32 (6/19)	6 (1/18)	27 (9/33)

Values represented as mean±SD and frequency (%).

*Ns vary because of missing data points.

NICU, neonatal intensive care unit; NS-SEC, national statistics socio-economic classification; SCBU, special care baby unit.

### Collection of inflammatory marker data

Data regarding blood levels of biomarkers were collected from laboratory reports and medical records. The most extreme values within the first 48 hours from the time of admission were recorded for CRP, total white cell count (WCC), neutrophils and lymphocytes. We included markers of inflammation-related coagulation anomalies (platelets and fibrinogen), and tissue hypoxia (lactate), in order to determine whether the aetiology of psychopathology was more likely to be neuroinflammatory in nature, or whether ischaemic/other processes may have contributed. These measures were tested in certified hospital laboratories at the relevant sites under nationally standardised procedures. The highest values in the first 48 hours of admission were recorded for all biomarkers except for platelets where lowest values were recorded (due to the association between enduring inflammation and a decrease in platelet count^18^).

### Psychiatric symptomatology

The following questionnaires were sent to children and their families 3–6 months after discharge from PICU to evaluate concurrent global psychiatric and PTSD risk.

#### Strengths and Difficulties Questionnaire (SDQ)

The parent-rated version of the SDQ was used to assess for psychiatric symptoms and risk of disorder, including emotional and behavioural difficulties.^19^ This questionnaire is validated for use in children aged 4–17 years. It consists of 25 items with answers on a 3-point scale. Overall risk for a psychiatric disorder is indicated by a total score higher than 17.

#### Impact of Events Scale (IES-8)

The child self-rated IES-8 was used to assess post-traumatic stress symptoms. This questionnaire is validated for use in children aged 8 years and above.^20^ This eight-item questionnaire rates frequency of PTSD symptoms in the last seven days on a 4-point scale. Overall risk for PTSD is indicated by a total score higher than 17.

### Statistical analysis

All statistical analyses were carried out using IBM SPSS Statistics V.24 for Windows. After removing outliers with values of >4 SD from the mean, all data sets were tested for normality using the Shapiro-Wilk test. χ^2^ tests for association were performed on categorical data, and Kruskal-Wallis tests were used to determine the differences in variables between the three illness subgroups. Partial correlation analyses (controlling for age on admission to PICU and gender) were performed on continuous data to determine the associations between biomarkers and questionnaire scores.

As this was an exploratory study, we report findings with p<0.05; however, results were also corrected for multiple comparisons using the Benjamini-Hochberg (BH) procedure. The BH correction is calculated through ranking p values in order, calculating a false discovery threshold by dividing the rank number by the number of multiple comparisons, and multiplying this for the false discovery rate, which we set at 10%. If a p value is smaller than the calculated threshold, significance is maintained; if equal or larger, significance is lost.^21^

## Findings

### Illness group variables

The full cohort was divided into illness groups as follows: sepsis (n=18), meningoencephalitis (n=19) and other illnesses (n=34). The composition of most demographic variables for all groups within the cohort was comparable (p>0.05 for age, socioeconomic status, ethnicity). Further data regarding these variables are included in table 1. Gender distribution varied between groups, with significantly more females in the sepsis group (χ^2^=8.230; df=2, p=0.016).

The three groups were matched for both previous health disorders (perinatal disorders, chronic medical conditions and general past health status) but varied according to illness severity characteristics of the patients’ acute illness (length of stay in PICU and PIM2). The mean (±SD) length of stay for the full cohort was 4.66±6.912 days and the mean (±SD) PIM2 score 11.42%±20.546%. Patients with sepsis were more likely to have a longer length of stay mean of 8.67±9.726 days (χ^2^=14.58; df=2, p=0.001) and worse PIM2 mean (±SD) score of 23.88%±30.987% (χ^2^=0.06; df=2, p=0.011) than patients from other subgroups.

### Biomarkers

Proportions of biomarkers were well-documented in clinical case records (>94%) except for three biomarkers: lymphocytes and fibrinogen were recorded in 70% (50/71) and lactate recorded in 52% (37/71). Both raw continuous values and categorical values (dividing biomarkers into categories of normal or abnormal) were used for analyses. Normal concentrations of these markers were evaluated according to standardised references ranges.^22^ Details of biomarker normal values, of means and of percentages of children with abnormal values in both the full cohort and individual illness groups are given in table 2.

**Table 2:** Inflammatory markers and frequency of abnormal results in full PICU cohort and patient subgroups

Biomarker (normal range)	Full cohort Mean±SD; % with abnormal values (n/N)	Meningoencephalitis Mean±SD; % with abnormal values (n/N)	Septic illness Mean±SD; % with abnormal values (n/N)	Other illnesses Mean±SD; % with abnormal values (n/N)
White cell count	18.54±9.85×10^6^	20.36±18.65×10^6^	18.74±10.50×10^6^	17.45±9.40×10^6^
(4.8–10.8×10^6^ cells/L)	79.7 (55/69)	77.8 (14/18)	83.3 (15/18)	78.8 (26/33)
Neutrophils	16.23±9.93×10^9^	19.14±11.98×10^9^	16.67±9.18×10^9^	14.27±8.78×10^9^
(3.0–5.8×10^9^ cells/L)	92.6 (63/68)	89.5 (17/19)	100 (17/17)	90.6 (29/32)
Lymphocytes	2.10±1.33×10^9^	1.96±0.86×10^9^	1.80±1.77×10^9^	2.37±1.25×10^9^
(1.2–3.4×10^9^ cells/L)	38 (19/50)	23.1 (3/13)	57.1 (8/14)	34.8 (8/23)
Platelets	214.39±106.22×10^9^	227.98±101.01×10^9^	129.56±81.82×10^9^	252.85±96.68×10^9^
(130–400×10^9^ cells/L)	31.4 (22/70)	21.1 (4/19)	100 (18/18)*	21.2 (7/33)
Fibrinogen	3.67±1.44	4.31±1.53	3.34±0.90	3.43±1.58
(2–4 g/L)	44 (22/50)	60 (9/15)	35.7 (5/14)	38.1 (8/21)
C reactive protein	144.02±114.34	137.52±121.32	220.36±102.98	90.05±84.51
(<5 mg/L)	85.1 (57/67)	89.5 (17/19)	100 (18/18)	73.3 (22/30)*
Lactate	2.59±1.89	1.56±0.84	3.67±2.53	2.32±1.19
(0.5–2.2 mmol/L)	40.5 (15/37)	20 (2/10)	53.8 (7/13)	42.9 (6/14)

Values represented as mean±SD and frequency. Percentages were calculated over the total number of observations in each category excluding missing data.

*Statistically significant associations BH corrected for multiple comparisons.

BH, Benjamini-Hochberg.

In the whole cohort, >80% of patients had abnormal levels of neutrophils (93%, 63/68), CRP (85%, 57/67) and total WCC (80%, 55/69). Biomarkers which were least likely to be abnormal were platelets (31%, 22/70) and lymphocytes (38%, 19/50). Lactate and fibrinogen were abnormal in 41% (15/37) and 44% (22/50), respectively. There were differences between illness groups, with significantly more abnormal values for platelets in patients with sepsis compared with all other patients (χ^2^=9.9; df=1; p=0.003). Comparing patients with other illnesses to all other patients, there were significantly fewer abnormal values for CRP (χ^2^=6.2; df=1; p=0.017, n=66). These statistical differences remained after correction for multiple comparisons. The remaining biomarkers did not show any significant differences between groups.

### Psychiatric outcomes

Full data sets for SDQ and IES-8 scores were available for 69 and 44 patients, respectively. Fewer patients completed the IES-8 due to the questionnaire only being validated for use in children>8 years of age. Table 3 shows that 20% (14/69) of children were determined to be at high risk for a global psychiatric disorder according to SDQ scores, and 32% (14/44) had IES-8 scores indicating a high risk for PTSD. Abnormal IES-8 scores were more likely to occur in sepsis (50%, 6/12), compared with meningoencephalitis (36%, 5/14) and other disorders (17%, 3/18). However, this difference did not reach statistical significance (χ^2^=3.831; df=2; p=0.147).

**Table 3:** Psychiatric questionnaire scores of paediatric intensive care unit cohort and illness subgroups

	Full cohort	Meningoencephalitis	Septic illness	Other illnesses
	Mean±SD% (n/N)	Mean±SD% (n/N)	Mean±SD% (n/N)	Mean±SD% (n/N)
SDQ				
Total score	11.28±6.42	12.11±7.47	12.62±6.65	10.09±5.63
High psychiatric risk	20.3 (14/69)	16.7 (3/18)	27.8 (5/18)	18.2 (6/33)
IES-8				
Total score	11.25±10.59	12.93±11.91	16.17±8.63	6.67±9.25
High PTSD risk	31.8 (14/71)	35.7 (5/14)	50 (6/12)	16.7 (3/18)

Values represented as mean±SD and frequency. Percentages were calculated over the total number of observations in each category excluding missing data.

IES-8, Impact of Events Scale; PTSD, post-traumatic stress disorder; SDQ, Strengths and Difficulties Questionnaire.

### Associations between biomarkers and psychiatric symptoms

#### Whole PICU group

In the whole group, a χ^2^ test for associations revealed a statistically significant link between abnormal SDQ scores and abnormal lymphocyte count (χ^2^=6.757, p=0.014, n=48), and this was maintained after applying the BH correction (table 4). A link between abnormal SDQ scores and abnormal lactate levels (χ^2^=5.250, p=0.029, n=35) was however rendered non-significant when the BH correction was applied. No significant association was found between abnormal biomarker levels and PTSD risk, as measured through the IES-8 questionnaire.

**Table 4:** Associations between abnormal biomarker levels and global psychiatric risk (Strengths and Difficulties Questionnaire (SDQ) score)

Abnormal biomarker	Psychiatric risk (SDQ)		χ^2^ (df); p values
	Low % (n/N)	High % (n/N)	
Total white cell count	80 (43/54)	85 (11/13)	0.167 (1); 0.514
Neutrophils	93 (49/53)	100 (13/13)	1.044 (1); 0.406
Lymphocytes	31 (12/39)	78 (7/9)	6.757 (1); 0.014*
Platelets	30 (16/54)	43 (6/14)	0.889 (1); 0.263
Fibrinogen	50 (20/40)	22 (2/9)	2.291(1); 0.126
C reactive protein	84 (43/51)	93 (13/14)	0.672 (1) 0.374
Lactate	28 (7/25)	70 (7/10)	5.250 (1); 0.029

Proportion of children with abnormal biomarkers according to high and low psychiatric risk.

*Statistically significant association BH corrected for multiple comparisons.

BH, Benjamini-Hochberg.

Partial correlation analysis (controlling for age on admission and gender) revealed no significant correlation between continuous biomarker data and questionnaire scores (SDQ and IES-8) in the whole group.

#### By illness subgroup

We considered whether there would be differences in the associations between inflammatory and related biomarkers and psychiatric outcomes in different illness groups (septic illness, meningoencephalitis and other critical illnesses) using partial correlational analysis. The results of this analysis can be seen in table 5.

**Table 5:** Associations between biomarkers and psychiatric questionnaire scores in the whole cohort and by illness group: Pearson partial correlation coefficients controlled for age on admission and gender

	White cell count (n=69)	Neutrophils (n=68)	Lymphocytes (n=50)	Platelets (n=70)	Fibrinogen (n=50)	CRP (n=67)	Lactate (n=37)
SDQ							
Full cohort (n=69)	−0.006	0.16	−0.021	−0.141	0.039	0.081	−0.024
Meningoencephalitis (n=18)	−0.165	0.256	**0.714**	−0.224	−0.175	−0.006	0.077
Septic illness (n=18)	−0.236	−0.162	−0.712*	−0.129	0.011	0.239	−0.226
Other illness (n=33)	0.047	0.109	−0.174	−0.039	0.299	−0.229	−0.051
IES-8							
Full cohort (n=44)	0.171	0.15	0.159	−0.301	0.201	0.193	−0.023
Meningoencephalitis (n=14)	−0.037	−0.085	**0.652**	−0.108	0.181	−0.557	−0.549
Septic illness (n=12)	0.156	0.164	0.092	−0.323	−0.418	0.823*	−0.379
Other illness (n=18)	0.238	0.301	−0.065	−0.17	−0.183	−0.204	−0.488

Bold values represent statistically significant association (p<0.05) before BH correction.

*Statistically significant associations BH corrected for multiple comparisons.

BH, Benjamini-Hochberg; CRP, C reactive protein; IES, Impact of Events Scale; SDQ, Strengths and Difficulties Questionnaire.

In children with septic illness, there was a negative association between SDQ scores and lymphocyte count (r=−0.712; p=0.009, n=14), and a positive association between IES-8 scores and CRP (r=0.823; p=0.006, n=11). Both associations were maintained after applying the BH correction.

In children with meningoencephalitis, a positive association between SDQ scores and lymphocyte count was identified (r=0.714; p=0.020, n=12), but this became non-significant following the BH correction.

In children with other critical illnesses, no associations were identified between biomarkers and psychiatric questionnaire scores.

#### Possible confounding effects of illness severity

It is possible that some of the significant links found between biomarkers and psychiatric outcomes were an indirect effect of illness severity on psychopathology rather than the effect of inflammation per se. To assess this possible confounding effect, the relationship in the whole PICU group between lymphocyte levels and measures of illness severity (PIM2 score and days spent in PICU) was investigated. This showed no significant correlation between lymphocytes and either PIM2 (r=−0.20; p=0.16, n=49) or length of stay (r=0.27; p=0.055, n=50). Subanalysis in the septic group showed the same result; neither lymphocyte nor CRP levels showed a significant correlation with measures of illness severity (lymphocytes vs PIM2 (r=0.024; p=0.935, n=14) and vs length of stay (r=0.16; p=0.58, n=14); CRP vs PIM2 (r=0.027; p=0.918, n=17) and vs length of stay (r=0.057; p=0.828, n=17)).

## Discussion

This exploratory study reports associations between inflammatory biomarkers and psychiatric outcomes in children following critical illness requiring admission to PICU. The results support the suggestion that inflammatory changes may be associated with psychiatric symptoms at 3–6 months following discharge, particularly for children who have suffered from sepsis. Measures of coagulation (platelets and fibrinogen) and of tissue hypoxia (lactate) did not show similar associations. This fact, together with the lack of significant associations between inflammatory markers and illness severity, suggests that at the cellular level presumed neuroinflammatory processes may be more relevant than systemic coagulopathy or whole-body ischaemia to adverse psychiatric outcome.

The overall psychiatric risk as measured by the SDQ was increased twofold in the whole PICU cohort compared with the expected general population rate (20% vs an expected rate of 10%), suggesting that alongside the illness-specific PTSD symptoms, approximately half of the SDQ abnormality rates might reflect de novo illness sequelae. As we have identified correlations between inflammatory biomarkers during acute illness and subsequent psychiatric difficulties, the proposed role of inflammation as a mediator in the development of psychopathology may partially explain this increase in prevalence.

### Inflammatory associations according to individual biomarkers

Among the biomarkers considered, lymphocyte count was found to be associated with SDQ scores, indicative of global psychiatric risk, and CRP with IES-8 scores, indicative of specific PTSD risk (as opposed to general psychiatric symptoms). While our study is not powered to detect specificity of particular biomarkers, the wider literature supports these two markers as being associated with the development of psychopathology.

#### Lymphocyte count

SDQ measured global psychiatric risk was associated with an abnormal lymphocyte count in the whole cohort, while in the septic group there was a significant negative correlation between SDQ scores and lymphocyte count.

Abnormal lymphocyte levels have been proposed as a potential biomarker for a number of psychiatric illnesses.^8^ A variety of abnormalities in lymphocyte profiles characterise different aspects of psychopathology and a low proliferative lymphocytic response, as detected in our septic group, has been shown particularly in individuals with anxiety disorders.^23^

The lymphocytic reaction represents part of the adaptive immune response to infectious pathogens and varies accordingly dependent on the nature of the pathogen. Sepsis is often the result of a severe bacterial infection. Bacterial pathogens, in contrast to viral, are known to deplete the lymphocyte count,^8 22^ therefore this finding may be a representation of the severity of the immune response to an overwhelming bacterial invasion in this group.

#### C reactive protein

CRP is a well-established marker for inflammation as levels correlate closely with the state of systemic inflammatory processes.^18^ Following an insult, pro-inflammatory cytokines (IL-1, IL-6 and TNF-α) trigger CRP production. Levels increase 4–6 hours from the inflammatory challenge and peak at 36–50 hours.^15^

In the septic group, there was a strong positive correlation between CRP levels and IES-measured PTSD symptom burden. This is in line with cross-sectional community studies, where patients with PTSD have been found to have approximately twice the odds of CRP elevations than those without PTSD.^9^ Furthermore, there may be a genetic driver with evidence supporting a single-nucleotide polymorphism within the CRP gene (rs1130864) being associated with higher PTSD rates in those who have experienced trauma.^24^

The mechanisms underlying this association rely on pro-inflammatory cytokines crossing the blood–brain barrier from the peripheral circulation and activating microglia. This in turn leads to alterations in neurotransmission and apoptosis.^25^ The pro-inflammatory state has also been demonstrated to significantly suppress hippocampal neurogenesis and high titres are associated with low hippocampal volume,^26^ a neuroanatomical trait that marks vulnerability to PTSD.^27^ It is possible that a rise in pro-inflammatory cytokines and subsequently CRP may be regarded as representing a biological response to trauma just as PTSD represents the parallel psychological response.

Overall the associations between inflammatory biomarkers and psychiatric outcome in our study are in keeping with findings from both preclinical and clinical research. These studies illustrate that the causal pathways are likely to be the induction of a pro-inflammatory phenotype by stress which leads to neuroendocrine stress hormone dysregulation and subsequent alterations in immune and inflammatory signalling.^28^

## Clinical implications

Given the exploratory nature of our study, replication on a larger scale is a prerequisite to determining its clinical relevance. If the results are replicated, potential clinical implications both in the acute setting and for follow-up are a possibility. Acutely, it is possible that focus towards reduction in systemic inflammation through use of anti-inflammatory medications may potentially reduce the development of psychiatric sequelae.^29 30^ To our knowledge, this hypothesis has not been tested observationally or experimentally in paediatric settings. Furthermore, it is possible that these findings may have prognostic value for certain children following discharge from PICU. In particular, they could be used to highlight those most likely to benefit from psychiatric follow-up and/or monitoring. In this case, predictive scores containing weighted lymphocyte and CRP values may be considered for general psychiatric problems and PTSD risk, respectively.

In conclusion, this study suggests that a relationship exists between inflammatory biomarkers during admission for critical illness and subsequent psychopathology in children following discharge from PICU. It further suggests that biological variabilities underlying different critical illnesses may differ in their effects on psychopathology due to their varied influence on neuroinflammatory processes. Considering previously established links between trauma, inflammation and psychopathology, this study may also have wider relevance beyond the critically ill population in understanding the patho-aetiology of trauma-related psychiatric sequelae.

### Limitations

Our main limitation lies in the possibility of the findings being the result of type 1 error given the sample size of the clinical data set, particularly when considering individual illness subtypes. It is also possible that some of our negative findings are less specific due to differences in the number of participants in the analyses undertaken due to missing data.

Previous studies investigating levels of inflammation in children following trauma have revealed considerably lower degrees of inflammation than those examined here due to the severe nature of acute illness in our cohort. In keeping with this, ceiling effects may have accounted for the lack of association for certain biomarkers since the majority of children displayed marked anomalies in some measures as values in these children are likely to represent maximal levels of abnormality. Furthermore, due to the cross-sectional nature of the study, we cannot rule out confounding from previous trauma or previous inflammatory abnormalities. However, as mentioned, any biomarker abnormalities resulting from these are likely to be low in degree.

There may be further additional confounders from the observational nature of the dataset in that the biomarker measurements, although standardised across centres, are designed specifically for clinical use. Furthermore, there may be group differences in residual physical health problems at follow-up and due to incomplete response from some families, it is conceivable that we may have missed some higher scoring individuals.

Finally, it must be stressed that the aetiology of psychopathology is multifactorial. Psychiatric risk is clearly heavily influenced by a number of psychosocial factors, including the psychological trauma of critical illness, but this study has looked solely at the biological aspects of critical illness.

Despite these weaknesses, this exploratory study has identified several positive associations which are in keeping with the existent literature. These deserve further investigation with the possible aim of identifying potential inflammatory biomarkers relating to poor psychiatric adjustment following critical illness.

### Future directions

Should these findings be reproduced following replication in a larger cohort, future studies to elucidate the molecular mechanisms underlying the relationships identified would be an important progression. It is possible that, given the close association between the inflammatory system and the HPA axis, a dysregulated glucocorticoid response (due to the experience of critical illness) and subsequent central effects of ‘inappropriate’ glucocorticoid levels may explain some of the outcomes. Further work would benefit from concurrent review of the acute and chronic stress responses in this way.
